# Supplementary material for: Heat adaptation of phage T7 under an extended genetic code
Source: Virus Evol. 2021 Dec 1;7(2):veab100. doi: 10.1093/ve/veab100 (PMC8923235; doi:10.1093/ve/veab100)
Supplement: veab100_Supp [file veab100_supp.zip › Sequencing_Analysis.zip!/Sequencing_Analysis/S2/evidence/JC_SIDE_2_41.html]

BRESEQ :: Evidence


| New junction evidence | | | | | | | | | | | |
| --- | --- | --- | --- | --- | --- | --- | --- | --- | --- | --- | --- |
|  | | seq id | position | reads (cov) | reads (cov) | score | skew | freq | annotation | gene | product |
| \* | ? | NC\_001604.1 | = 281 | 5233 (1.110) | 1199 (0.240) +TAAAGACCAGACC | 21/508 | 41.8 | 17.7% | intergenic (–/–) | *–/–* | –/– |
|  | ? | NC\_001604.1 | 282 = | 5794 (1.090) |  |  |  |  | intergenic (–/–) | *–/–* | –/– |
| Rejected: Coverage evenness skew score above cutoff. | | | | | | | | | | | |
| Rejected: Frequency below/above cutoff threshold. | | | | | | | | | | | |

Only 100 of 6999 total aligned reads displayed.  

`‑‑‑‑‑‑‑‑‑‑‑‑‑TCTCACAGTGTACGGACCTAAAGTTCCCCCATAGGGGGTACCTAAAG‑CCCAGCCAATCACCTAAAGTCAACCTTCGGTTGACCTTGAGGGTT‑CCCTAAGGGTTGGGGATGACCCTTGGGTTTGTCTTTGGGTGTTACCTTGAGTGTCTCTCTGTGT‑CCC‑TATCTGTTACAGTCTCCTAAAGTATCCTCCTAAAGTCACCTCCTAACGTCCATCCTAAAGCCAACACCTAAAGCCTACACCTAAAGACCCATCAAGTCAACGCCTATCTTAAAGTTTAAACATAAAGACCAGACCTAAAGACCAGACCTAAAGACACTACATAAAGACCAGACCTAAAGACGCCTTGTTGTTAGCCATAAAGTGATAACCTTTAATCATTGTCTTTATTAATACAACTCACTATAAGGAGAGACAACTTAAAGAGACTTAAAAGATTAATTTAAAATTTATCAAAAAGAGTATTGACTTAAAGTCTAACCTATAGGATACTTACAGCCATCGAGAGGGACACGGCGAATAGCCATCCCAATCGACACCGGGGTCAACCGGATAAGTAGACAGCCTGATAAGTCGCACGAAAAA  >  NC_001604.1/1‑582`  

                                                                                                                                                                                                                                                                                                          `|`                                                                                                                                                                                                                                                                                                              
`gagatgtgtataagagacaggtgtACGGACCTAAAGTTCCCCCATAGGGGGTACCTAAAG‑CCCAGCCAATAACCTAAAGTCAACCTTCGGTTGACCTTGAGGGTT‑CCCTAAGGGTTGGGGATGACCCTTGGGTTTGTCTTTGGGTGTTACCTTGAGTGTCTCTCTGTGT‑CCC‑TATCTGTTACAGTCTCCTAAAGTATCCTCCTAAAGTCACCTCCTAACGTCCATCCTAAAGCCAACACCTAAAGCCTACACCTAAAGACCCATCAAGTCAACGCCTATCTTAAAGTTTAAACAtaaagac                                                                                                                                                                                                                                                                                                        <  1:214662/281‑1 (MQ=255)`  
              `agacaggtgtACGGACCTAAAGTCCCCCCATAGGGGGTACCTAAAG‑CCCAGCCAATCACCTAAAGTCAACCTTCGGTTGACCTTGAGGGTT‑CCCTAAGGGTTGGGGATGACCCTTGGTTTTGTCTTTGGGTGTTACCTTGAGTGTCTCTCTGTGT‑CCC‑GATCTGTTACAGTCTCCTAAAGTATCCTCCTAAAGTCACCTCCTAACGTCCATCCTAAAGCCAACACCTAAAGCCTACACCTAAAGACCCATCAAGTCAACGCCTATCTTAAAGTTTAAACAtaaagaccagacctaaagacc                                                                                                                                                                                                                                                                                          <  1:724178/295‑1 (MQ=255)`  
                  `caGTGTACGGACCTAAAGTTCCCCCATAGGGGGTACCTAAAG‑CCCAGCCAATCACCTAAAGTCAACCTTCGGTTGACCTTGAGGGTT‑CCCTAAGGGTTGGGGATGACCCTTGGGTTTGTCTTTGGGTGTTACCTTGAGTGTCTCTCTGTGT‑CCC‑TATCTGTTACAGTCTCCTAAAGTATCCTCCTAAAGTCACCTCCTAACGTCCATCCTAAAGCCAACACCTAAAGCCTACACCTAAAGACCCATCAAGTCAACGCCTATCTTAAAGTTTAAAAAtaaagaccagacctaacgaccagac                                                                                                                                                                                                                                                                                      >  1:818723/1‑301 (MQ=255)`  
                   `ggtgtACGGACCTAAAGTTCCCCCATAGGGGGTACCTAAAG‑CCCAGCCAATCACCTAAAGTCAAACTTCGGTTGACCTTGAGGGTT‑CCCTAAGGGTTGGGGATGACCCTTGGGTTTGTCGTTGGGTGTTACCTTGAGTGTCTCTCTGTGT‑CCC‑TATCTGTTACAGTCTCCTAAAGTATCCTCCTAAAGTCACCTCCTAACGTCCATCCTAAAGCCAACACCTAAAGCCTACACCTAAAGACCCATCAAGTCAACGCCTATCTTAAAGTTTAAACAtaaagaccagacctaaagaccagacc                                                                                                                                                                                                                                                                                     <  1:483455/300‑1 (MQ=255)`  
                    `gtgtACGGACCTAAAGTTCCCCCATAGGGGGTACCTAAAG‑CCCAGCCAATCACCTAAAGTCAACCTTCGGTTGACCTTGAGGGTT‑CCCTAAGGGTTGGGGATGACCCTTGGGTTTGTCTTTTGGTTTTACCTTGAGTGTCTCTCTGTGT‑CCC‑TATCTGTTACAGTCTCCTAACGTATCCTCCTAAAGTCACCTCCTAACGTCCATCCTAACGCCAACACCTAAAGCCTACACCTAAAGACCCATCAAGTCAACGCCTATCTTAACGTTTAAACATcaagaccaaccctaaaacccagacct                                                                                                                                                                                                                                                                                    >  1:423154/1‑301 (MQ=255)`  
                    `gtgtACGGACCTAAAGTTCCCCCATAGGGGGTACCTAAAG‑CCCAGCCAATCACCTAAAGTCAACCTTCGGTTGACCTTGAGGGTT‑CCCTAAGGGTTGGGGATGACCCTTGGGTTTGTCTTTGGGTGTTACCTTGATTGTCTCTCTGTGT‑CCC‑TATCTGTTCCAGTCTCCTAAAGTATCCTCCTAAAGTCACCTCCTAACGTCCATCCTAAAGCCAACACCTAAAGCCTACACCTAAAGACCCATCAAGTCAACGCCTATCTTAAAGTTTAAACAtaaagaccagacctaaagctgtctctt                                                                                                                                                                                                                                                                                    >  1:438801/1‑292 (MQ=255)`  
                    `gtgtACGGACCTAAAGTTCCCCCATAGGGGGTACCTAAAG‑CCCAGCCAATCACCTAAAGTCAACCTTCGGTTGACCTTGAGGGTT‑CCCTAAGGGTTGGGGATGACCCTTGGGTTTGTCTTTGGGTGTTACCTTGAGTGTCTCTCTGTGT‑CCC‑TATCTGTTACAGTCTCCTAAAGTATCCTCCTAAAGTTACCTCCTAACGTCCATCCTAAAGCCAACACCTAAAGCCTACACCTAAAGACCCATCAAGTCAACGCCTATCTTAAAGTTTACACATaaagaccagacctaaagaccagacct                                                                                                                                                                                                                                                                                    >  1:650591/1‑301 (MQ=255)`  
                    `gtgtACGGACCTAAAGTTCCCCCATAGGGGGTACCTAAAG‑CCCAGCCAATCACCTAAAGTCAACCTTCGGTTGACCTTGAGGGTT‑CCCTAAGGGTTGGGGATGACCCTTGGGTTTGTCTTTGGGTGTTACCTTGAGTGTCTCTCTGTGT‑CCC‑TATCTGTTACAGTCTCCTAAAGTATCCTCCTAAAGTCACCTCCTAACGTCCATCCTAAAGCCAACACCTAAAGCCTACACCTAAAGACCCATCAAGTCAACGCCTATCTTAAAGTTTAAACATaaagaccagacctaaagaacagacct                                                                                                                                                                                                                                                                                    >  1:185755/1‑301 (MQ=255)`  
                    `gtgtACGGACCTAAAGTTCCCCCATAGGGGGTACCTAAAG‑CCCAGCCAATCACCTAAAGTCAACCTTCGGTTGACCTTGAGGGTT‑CCCTAAGGGTTGGGGATGACCCTTGGGTTTGTCTTTGGGTGTTACCTTGAGTGTCTCTCTGTGT‑CCC‑TATCTGTTACAGTCTCCTAAAGTATCCTCCTAAAGTCACCTCCTAACGTCCATCCTAAAGCCAACACCTAAAGCCTACACCTAAAGACCCATCAAGTCAAAGCCTATCTTAAAGTTTAAACATaaagaccagacctaaagaccagacct                                                                                                                                                                                                                                                                                    >  1:202147/1‑301 (MQ=255)`  
                    `gtgtACGGACCTAAAGTTCCCCCATAGGGGGTACCTAAAG‑CCCAGCCAATCACCTAAAGTCAACCTTCGGTTGACCTTGAGGGTT‑CCCTAAGGGTTGGGGATGACCCTTGGGTTTGTCTTTGGGTGTTACCTTGAGTGTCTCTCTGTGT‑CAC‑TATCTGTTACAGTCTCCTAAAGTATCCTCCTAAAGTCACCTCCTAACGTCCATCCTAAAGCCAACACCTAAAGCCTACACCTAAAGACCCATCAAGTCAACGCCTATCTTAAAGTTTAAACATaaagaccagacctaaagaccagacct                                                                                                                                                                                                                                                                                    >  1:581687/1‑301 (MQ=255)`  
                      `gtACGGACCTAAAGTTCCCCCATAGGGGGTACCTAAAG‑CCCAGCCAATCACCTAAAGTCAACCTTCGGTTGACCTTGAGGGTT‑CCCTAAGGGTTGGGGATGACCCTTGGGTTTGTCTTTGGGTGTTACCTTGAGTGTCTCTCTGTGT‑CCC‑TATCTGTTCCAGTCTCCTAAAGTATCCTCCTAAAGTCACCTCCTAACGTCCATCCT‑AAGCCAACACCTAAAGCCTACACCTAAAGACCCATCAAGTCAACGCCTATCTTAAAGTTTAAACAtaaagaccagacctaaagaccagaccctgt                                                                                                                                                                                                                                                                                 >  1:444636/1‑297 (MQ=255)`  
                      `gtACGGACCTAAAGTTCCCCCATAGGGGGTACCTAAAG‑CCCAGCCAATCACCTAAAGTCAACCTTCGGTTGACCTTGAGGGTT‑CCCTAAGGGTTGGGGATGACCCTTGGGTTTGTCTTTGGGTGTTACCTTGAGTGTCTCTCTGTGT‑CCC‑GATCTGTTACAGTCTCCTAAAGTATCCTCCTAAAGTCACCTCCTAACGTCCATCCTAAAGCCAACACCTAAAGCCTACACCTAAAGACCCATCAAGTCAACGCCTATCTTAAAGTTTAAACATAAagaccagacctaaagaccagacctaa                                                                                                                                                                                                                                                                                  >  1:605686/1‑301 (MQ=255)`  
                         `gggcccaaaaGTTCCCCCAAAGGGGGT‑CCTAAAGCCCCAGCCAACCCCCTAAAGT‑AACCCTTGGTTTGCCTTTGGGGTT‑TCCTAAGGGTTGGGGATGACCCTTGGGTTTGTCTTTGGGTGTTACCTTGAGTGTCTCTCTGTGT‑CCC‑TATCTGTTACAGTCTCCTAAAGTATCCTCCTAAAGTCACCTCCTAACGTCCATCCTAAAGCAAACACCTAAAGCCTACACCTAAAGACCCATCAAGTCAACGCCTATCTTAAAGTTTAAACATAAAGACcagacctaaagaccagacctaaagac                                                                                                                                                                                                                                                                              <  1:429899/294‑1 (MQ=37)`  
                           `gACCTAAAGTTCCCCCATAGGGGGTACCTAAAG‑CCCAGCCAATCACCTAAAGTCAACCTTCGGTTGACCTTGATGGTT‑CCCTAAGGGTTGGGGATGACCCTTGGGTTTGTCTTTGGGTGTTACCTTGAGTGTCTCTCTGTGT‑CCC‑TATCTGTTGCAGTCTCCTAAAGTATCCTCCTAAAGTCACCTCCTAACGTCCATCCTCAAGCCAACACCTAAAGCCTACACCTAAAGACCCATCAAGTCAACGCCTATCTTAAAGTTTAAACCTAAAGACCAAACCTAAAGACCAGACCtacataca                                                                                                                                                                                                                                                                             >  1:537489/1‑301 (MQ=255)`  
                           `gACCTAAAGTTCCCCCATAGGGGGTACCTAAAG‑CCCAGCCAATCACCTAAAGTCAACCTTCGGTTGACCTTGAGGGTT‑CCCTAAGGGTTGGGGATGACCCTTGGGTTTGTCTTTGGGTGTTACCTTGAGTGTCTCTCTGTGT‑CCC‑TATCTGTTACAGTCTCCTAAAGTATCCTCCTAAAGTCACCTCCTAACGTCCATCCTAAAGCCAACACCTAAAGCCTACACCTAAAGACCCATCAAGTCAACGCCTATCTTAAAGTTTAAACATAAAGCCCCGACCTAAAGACCCGACCTAAAGaca                                                                                                                                                                                                                                                                             >  1:702217/1‑301 (MQ=255)`  
                           `gACCTAAAGTTCCCCCATAGGGGGTACCTAAAG‑CCCAGCCAATCACCTAAAGTCAACCTTCGGTTGACCTTGAGGGTT‑CCCTAAGGGTTGGGGATGACCCTTGGGTTTGTCTTTGGGTGTTACCTTGAGTGTCTCTCTGTGT‑CCC‑TATCTGTTACAGTCTCCTAAAGTATCCTCCTAAAGTCACCTCCTAACGTCCATCCTAAAGCCAACACCTAAAGCCTACACCTAAAGACCCATCAAGTCAACGCCTATCTTAAAGTTTAAACATAAAGACCAGACCTAAAGACCAGACCTAAAGaca                                                                                                                                                                                                                                                                             >  1:740357/1‑301 (MQ=255)`  
                           `gACCTAAAGTTCCCCCATAGGGGGTACCTAAAG‑CCCAGCCAATCACCTAAAGTCAACCTTCGGTTGACCTTGAGGGTT‑CCCTAAGGGTTGGGGATGACCCTTGGGTTTGTCTTTGGGTGTTACCTTGAGTGTCTCTCTGTGT‑CCC‑TATCTGTTACAGTCTCCTAAAGTATCCTCCTAAAGTCACCTCCTAACGTCCATCCTAAAGCCAACACCTAAAGCCTACACCTAAAGACCCATCAAGTCAACGCCTATCTTAAAGTTTAAACATAAAGACCAGACCTAAAGACCAAACCTAACGaca                                                                                                                                                                                                                                                                             >  1:748808/1‑301 (MQ=255)`  
                           `gACCTAAAGTTCCCCCATAGGGGGTACCTAAAG‑CCCAGCCAATCACCTAAAGTCAACCTTCGGTTGACCTTGAGGGTT‑CCCTAAGGGTTGGGGATGACCCTTGGGTTTGTCTTTGGGTGTTACCTTGAGTGTCTCTCTGTGT‑CCC‑GGTCTGTTACAGTCTCCTAAAGTATCCTCCTAAAGTCACCTCCTAACGTCCATCCTAAAGCCAACACCTAAAGCCTACACCTAAAGACCCATCAAGTCAACGCCTATCTTTAAGTTTAAACATAAAGACCAGACCTAAAGACCAGACCTAAAGaca                                                                                                                                                                                                                                                                             >  1:335129/1‑301 (MQ=255)`  
                           `gACCTAAAGTTCCCCCATAGGGGGTACCTAAAG‑CCCAGCCAATCACCTAAAGTCAACCTTCGGTTGACCTTGAGGGTT‑CCCTAAGGGTTGGGGATGACCCTTGGGTTTGTCTTTGGGTGTTACCTTGAGTGTCTCTCTGTGT‑CCC‑GATCTGTTACAGTCTCCTAAAGTATCCTCCTAAAGTCACCTCCTAACGTCCATCCTAAAGCCAACACCTAAAGCCTACACCTAAAGACCCATCAAGTCAACGCCTATCTTAAAGTTTAAACATAAAGACcagacctaaagaccagacctaaagacc                                                                                                                                                                                                                                                                             >  1:278962/1‑300 (MQ=255)`  
                                      `cctccATAGGGGGTACCGTAAG‑ACCCGCCAATCTCCTAAAGTCAACCTTCGGTTGACCTTGAGGGTT‑CCCTGAGGGTTGGGGATGACCCTTGGGTTTGTCTTTGGGTGTTACCTTGAGTGTCTCTCTGTGT‑CCC‑GATCTGTTTCAGTCACCTAAAGTATCCTCCTAAAGTCACCTCCTAACGTCCATCCTAAAGCCAACACCTAAAGCCTACACCTAAAGACCCATCAAGTCAACGCCTATCTTAAAGTTTAAACATAAAGCCCAGACCTAAAGACCAGACCTAAAGACACTACATAAaga                                                                                                                                                                                                                                                                  <  1:300346/298‑1 (MQ=37)`  
                                       `ccccATAGTGGGTACCTAAAG‑CCCAGCCAATCACCTAAAGTCAACCTTCGGTTGACCTTGAGGGTT‑CCCTAAGGGTTGGGGATGACCCTTGGGTTTGTCTTTGGGTGTTACCTTGAGTGTCTCTCTGTGT‑CCC‑TATCTGTTACAGTCTCCTAAAGTATCCTCCTAAAGTCACCTCCTAACGTCCATCCTAAAGCCAACACCTAAAGCCTACACCTAAAGACCCATCAAGTCAACGCCTATCTTAAAGTTTAAACATAAAGACCAGACCTAAAGACCAGACCTAAAGACACTACATAAagac                                                                                                                                                                                                                                                                 <  1:465332/301‑1 (MQ=255)`  
                                       `ccccATAGGGGGTACCTAAAG‑CCCAGCCAATCACCTAAAGTCAACCTTCGGTTGACCTTGAGGGTT‑CACTAAGGGTTGGGGATGACCCTTGGGTTTGTCTTTGGGTGTTACCTTGAGTGTCCCTCTGTGT‑CCC‑TATCTGTTACAGTCTCCTAAAGTATCCTCCTAAAGTCACCTCCTAACGTCCATCCTAAAGCCAACACCTAAAGCCTACACCTAAAGACCCATCAAGTCAACGCCTATCTTAAAGTTTAAACATAAAGACCAGAACTAAAGACCAGACCTAAAGACACTACATAAagag                                                                                                                                                                                                                                                                 >  1:567467/1‑300 (MQ=255)`  
                                        `cccATAGGGGGTACCTAAAG‑CCCAGCCAATCACCTAAAGTCAACCTTCGGTTGACCTTGAGGGTTCCCCTAAGGGTTGGGGATGACCCTTGGGTTTGTCTTTGGGTGTTACCTTGAGTGTCTCTCTGTGT‑CCC‑TATCTGTTACAGTCTCCTAAAGTATCCTCCTAAAGTCACCTCCTAACGTCCATCCTAAAGCCAACACCTAAAGCCTACACCTAAAGACCCATCAAGTCAACGCCTATCTTAAAGTTTAAACATAAAGACCAGACCTAAAGACCAGACCTAAAGCCACTACATAAagac                                                                                                                                                                                                                                                                 >  1:590234/1‑301 (MQ=255)`  
                                        `cccATAGGGGGTACCTAAAG‑CCCAGCCAATCACCTAAAGTCAACCTTCGGTTGACCTTGAGGGTT‑CCCTAAGGGTTGGGGATGACCCTTGGGTTTGTCTTTGGGTGTTACCTTGAGTGTCTCTCTGTGT‑CCC‑TATCTGTTACAGTCTCCTAAAGTATCCTCCTAAAGTCACCTCCTAACGTCCATCCTAAAGCCAACACCTAAAGCCTACACCTAAAGACCCATCAAGTCAACGCCTATCTTAAAGTTTAAACATAAAGACCAGACCTAAAGACCAGACCTAAAGACACTACATAAagacc                                                                                                                                                                                                                                                                >  1:45797/1‑301 (MQ=255)`  
                                          `cATAGGGGGTACCTAAAG‑CCCAGCCAATCACCTAAAGTCAACCTTCGGTTGACCTTGAGGGTT‑CCCTAAGGGTTGGGGATGACCCTTGGGTTTGTCTTTGGGTGTTACCTTGAGTGTCTCTCTGTGT‑CCC‑TATCTGTTACAGTCTCCTAAAGTATCCTCCTAAAGTCACCTCCTAACGTCCATCCTAAAGCCAACACCTAAAGCCTACACCTAAAGACCCATCAAGTCAACGCCTATCTTAAAGTTTAAACATAAAGACCAGACCTAAAGACCAGACCTAAAGACACTACATAAcgaccaa                                                                                                                                                                                                                                                              >  1:498712/1‑300 (MQ=255)`  
                                              `gggggTACCTAAAG‑CCCAGCCAATCACCTAAAGTCAACCTTCGGTTTACCTTGAGGGTT‑CCCTAAGGGTTGGAGATGACCCTTGGGTTTGTCTTTGGGTGTTACCTTGAGTGTCTTCCTGTGT‑CCC‑AATCTGTTACAGTCTCCTAAAGAATCCTCCTAAAGTCACCTCCTAACGACCATCCTAAAGCCAACACCTAAAGTCTACACCTAAAGATCAACCAAGTCAACGCCTATCTTCAAGTTTAAACATAAAGACCAGACCTAAAGACCAACCCTCAAGACACTCCATACagctgtctctt                                                                                                                                                                                                                                                          >  1:393407/1‑292 (MQ=25)`  
                                                  `gTACCTAAAG‑CCCAGCCAATCACCTAAAGTCAACCTTCGGTTGACCTTGAGGGTT‑CCCTAAGGGTTGGGGATGACCCTTGGGTTTGTCTTTGGGTGTTACCTTGAGTGTCTCTCTGTGT‑CCC‑GATCTGTTACAGTCTCCTAAAGTATCCTACTAAAGTCACCTCCTAACGTCCATCCTAAAGCCAACACCTAAAGCCTACACCTAAAGACCCATCAAGTCAACGCCTATCTTAAAGTTTAAACATAAAGACCAGACCTAAAGACCAGACCTAAAGACACTACATAAAGACCAGACCTAAAg                                                                                                                                                                                                                                                      <  1:635913/301‑1 (MQ=255)`  
                                                    `aCCTAAAG‑CCCAGCCAATCACCTAAAGTCAACCTTCGGTTGACCTTGAGGGTT‑CCCTAAGGGTTGGGGATGACCCTTTGGTTTGTCTTTGGGTGTTACCTTGAGTGTCTCTCTGAGT‑CCC‑TATCTGTTACAGTCTCCTAAAGTATCCTCCTAAAGTCACCTCCTAACGTCCATCCTAAAGCCAACACCTAAAGCCTACACCTAAAGACCCATCAAGTCACCGCCTATCTTAAAGTTTAAACATAAAGACCAGACCTAAAGACCAGACCTACAGCCACTCCATAAAACCCAGACCTAAAGAc                                                                                                                                                                                                                                                    >  1:328351/1‑301 (MQ=255)`  
                                                    `aCCTAAAG‑CCCAGCCAATCACCTAAAGTCAACCTTCGGTTGACCTTGAGGGTT‑CCCTAAGGGTTGGGGATGACCCTTGGGTTTGTCTTTGGGTGTTACCTTGAGTGTCTCTCTGTGT‑CCC‑GATCTGTTACAGTCTCCTAAAGTATCCTCCTAAAGTCACCTCCTAACGTCCATCCTAAAGCCAACACCTAAAGCCTACACCTAAAGACCCATCAAGTCAACGCCTATCTTAAAGTTTAAACATAAAGACCAGACCTAAAGACCAGACCTAAAGACACTACATAAAGACCAGACCTAAAGAc                                                                                                                                                                                                                                                    <  1:122289/301‑1 (MQ=255)`  
                                                     `ccTAAAG‑CCCAGCCAATCACCTAAAGTCAACCTTCGGTTGACCTTGAGGGTT‑CCCTAAGGGTTGGGGATGATCCTTGGGTTTGTCTTTGGGTGTTACCTTGAGTGTCTCTCTGTGT‑CCC‑TATCTGTTCCAGTCTCCTAAAGTATCCTCCTAAAGTCACCTCCTAACGTCCATCCTAAAGCCAACACCTAAAGCCTACACCTAAAGACCCATCAAGTCAACGCCTATCTTAAAGTTTAAACATAAAGACCAGACCTAAAGACCAGACCTAAAGACACTACATAAAGACCAGACCTAAAGACg                                                                                                                                                                                                                                                   >  1:505414/1‑301 (MQ=255)`  
                                                                         `ccTAAAGTCAACCTTCGGTTGACCTTGAGGGTT‑CCCTAAGGGTTGGGGATGACCCTTGGGTTTGTCTTTGGGTGTTACCTTGAGTGTCTCTCTGTGT‑CCC‑GATCTGTTACAGTCTCCTAAAGTATCCTCCTAAAGTCACCTCCTAACGTCCATCCTAAAGCCAACACCTAAAGCCTACACCTAAAGACCCATCAAGTCAACGCCTATCTTAAAGTTTAAACATAAAGACCAGACCTAAAGACCAGACCTAAAGACACTACCTAAAGACCAGACCTAAAGACGCCTTGTTGTTAGTCATaaa                                                                                                                                                                                                                                >  1:521786/1‑301 (MQ=255)`  
                                                                          `cTAAAGTCAACCTTCGGTTGACCTTGAGGGTT‑CCCTAAGGGTTGGGGATGACCCTTGGGTTTGTCTTTGGGTGTTACCTTGAGTGTCTCTCTGTGT‑CCC‑TATCTGTTACAGTCTCCTAAAGTATCCTCCTAAAGTCACCTACTAACGTCCATCCTAAAGCCAACACCTAAAGCCTACACCTAAAGACCCATCAAGTCAACGCCTATCTTAAAGTTTAAACATAAAGAACAGACCTAAAGACCAGACCTAAAGACACTACATAAAGACCAGACCTAAAGACGCCTTGTTGTTAGCCATAAAg                                                                                                                                                                                                                               <  1:686208/301‑1 (MQ=255)`  
                                                                               `gTCAACCTTCGGTTGACCTTGAGGGTT‑CCCTAAGGGTTGGGGATGACCCTTGGGTTTGTCTTTGGGTGTTACCTTGAGTGTCTCTCTGTGT‑CCC‑TATCTGTTACAGTCTCCTAAAGTATCCTCCTAAAGTCACCTCCTAACGTCCATTCTAAAGCCAACACCTAAAGCCTACACCTAAAGACCCATCAAGTCAACGCCTATCTTAAAGTTTAAACATAAAGACCAGACCTACAGCCACGACCTAAAGACACTACATACAGACCAGACCTAAAGCCGCCTTGTTGTTAGCCATAAAGTGact                                                                                                                                                                                                                          >  1:320861/1‑299 (MQ=255)`  
                                                                                   `aCCTTCGGTTGACCTTGAGGGTT‑CCCTAAGGGTTGGGGATGACCCTTGGGTTTGTCTTTGGGTGTTACCTTGAGTGTCTCTCTGTGT‑CCC‑TATCTGTTACAGTCTCCTAAAGTATCCTCCTAAAGTCACCTCCTAACGTCCATCCTAAAGCCAACACCTAAAGCCTACACCTAAAGACCCATCAAGTCAACGCCTATCTTAAAGTTTAAACATAAAGACCAGACCTAAAGACCAGACCTAAAGACACTACATAAAGACCAGACCTAAAGACGCCTTGTTGTTAGCCATAAAgctgtctctt                                                                                                                                                                                                                      >  1:350782/1‑292 (MQ=255)`  
                                                                                        `cGGTTGACCTTGGGGGTT‑CCCGAAGGGTTGGGGATGACCCTTGGGTTTGTCTTTGGGTGTTACCTTGAGTGTCCCTCTGTGT‑CCC‑GCTCTGTTACAGTCTCCTAAAGTATCCTCCTAAAGTCACCTCCTAACGTCCATCCTAAAGCCAACACCTAAAGCCTACACCTAAAGACCCATCAAGTCAACGCCTATCTTAAAGTTTAAACAT‑AAGACCAGACCTAAAGACCAGACCTAAAGACACTACATAAAGACCAGACCTAAAGACGCCTTGTTGTTAGCCATAAAGTGATAACCTTTAATc                                                                                                                                                                                                                <  1:169901/301‑1 (MQ=255)`  
                                                                                         `ggTTGACCTTGAGGGTT‑CCCTAAGGGTTGGGGATGACCCTTGGGTTTGTCTTTGGGTGTTACCTTGAGTGTCTCTCTGTGT‑CCC‑TATCTGTTACAGTCTCCTAAAGTATCCTCCTAAAGTCACCTCCTAACGTCCATCCTAAAGCCAACACCTAAAGCCTACACCTAAAGACCCATCAAGTCAACGCCTATCTTAAAGTTTAAACATAAAGACCAGACCTAAAGACCAGACCTAAAGACACTACATAAAGACCAGACCTAAAGACGCCTTGTTGTTAGCCATAAAGTGATAACCTTTAATc                                                                                                                                                                                                                <  1:643270/301‑1 (MQ=255)`  
                                                                                         `ggTTGACCTTGAGGGTT‑CCCTAAGGGTTGGGGATGACCCTTGGGTTTGTCTTTGGGTGTTACCTTGAGTGTCTCTCTGTGT‑CCC‑GATCTGTTACAGTCTCCTAAAGTATCCTCCTAAAGTCACCTCCTAACGTCCATCCTAAAGCCAACACCTAAAGCCTACACCTAAAGACCCATCAAGTCAACGCCTATCTTAAAGTTTAAACATAAAGACCAGACCTAAAGACCAGACCTAAAGACACTACATAAAGACCAGACCTAAAGACGCCTTGTTGTTAGCCATAAAGTGATAACCTTTAATc                                                                                                                                                                                                                <  1:757147/301‑1 (MQ=255)`  
                                                                                         `agacagccTTGAGGTTT‑CCCTAAGGGGTGGGGATGACCCTTGGGTTTGTCTTTGGGTGTTACCTTGAGTGTCTCTATGTGT‑CCC‑GATCTGTTACAGTCTCCTAAAGTATCCTCCTAAAGTCACCTCCTAACGTCCATCCTAAAGCCAACACCTAAAGCCTACACCTAAAGACCCATCAAGTCAACGCCTATCTTAAAGTTTAAACATAAAGACCAGACCTAAAGACCAGACCTAAAGACACTACATAAAGACCAGACCTAAAGACGCCTTGTTGTTAGCCATAAAGTGATAACCTTTAATc                                                                                                                                                                                                                <  1:380577/295‑1 (MQ=255)`  
                                                                                         `aagagacagtGAGGGTT‑CCCTAAGGGTTGGGGATGACCCTTGGGTTTGTCTTTGGGTGTTACCTTGAGTGTCTCTCTGTGT‑CCC‑TATCTGTTACAGTCTCCTAAAGTATCCTCCTAAAGTCACCTCCTAACGTCCATCCTAAAGCCAACACCTAAAGCCTACACCTAAAGACCCATCAAGTCAACGCCTATCTTAAAGTTTAAACATAAAGACCAGACCTAAAGACCAGACCTAAAGACACTACATAAAGACCAGACCTAAAGACGCCTTGTTGTTAGCCATAAAGTGATAACCTTTAATc                                                                                                                                                                                                                <  1:558882/292‑1 (MQ=255)`  
                                                                                          `gTTGACCTTGAGGGTT‑CCCTAAGGGTTGGGGATGACCCTTGGGTTTGTCTTTGGGTGTTACCTTGAGTGTCTCTCTGTGT‑CCC‑TATCTGTTACAGTCTCCTAAAGTATCCTCCTAAAGTCACCTCCTAACGTCCATCCTAAAGCCAACACCTAAAGCCTACACCTAAAGACCCATCAAGTCAACGCCTATCTTAAAGTTTAACCATAAAGACCAGACCTAAAGACCAGACCTAAAGACACTACATAAAGACCAGACCTAAAGACGCCTTGTTGTTAGCCATAAATTGATAACCtctgtctc                                                                                                                                                                                                               >  1:811109/1‑294 (MQ=255)`  
                                                                                          `atgtgtataagagacagcccGAAGGGGTGGGGATGACCCTTGGGTTTGTCTTTGGGTGTGACCTTGAGTGTCTCTCTGTGT‑CCC‑GATCTGTTACAGTCTCCTAAAGTATCCTCCTAAAGTCACCTCCTAACGTCCATCCTAAAGCCAACACCTAAAGCCTACACCTAAAGACCCATCAAGTCAACGCCTATCTTAAAGTTTAAACATAAAGACCAGACCTAAAGACCAGACCTAAAGACACTACATAAAGACCAGACCTAAAGACGCCTTGTTGTTAGCCATAAAGTGATAACCTTTAATc                                                                                                                                                                                                                <  1:620065/284‑1 (MQ=255)`  
                                                                                             `gACCTTGAGGGTT‑CTCTAAGGGTTGGGGATGACCCTTGGGTTTGTCTTTGGGTGTTACCTTGAGTGTCTCTCTGTGT‑CCC‑TATCTGTTACAGTCTCCTAAAGTATCCTCCTAAAGTCACCTCCTAACGTCCATCCTAAAGCCAACACCTAAAGCCTACACCTAAAGACCCATCAAGTCAACGCCTATCTTAAAGTTTAAACATAAAGACCAGACCTAAAGACCAGACCTAAAGACACTACATAAAGACCAGACCTAAAGACGCCTTGTTGTTAGCCATAAAGTGATAACCTTTAATCAttt                                                                                                                                                                                                            >  1:774442/1‑300 (MQ=255)`  
                                                                                             `gACCTTGAGGGTT‑CCCTAAGGGTTGGGGATGACCCTTGGGTTTGTCTTTGGGTGTTACCTTGAGTGTCTCTCTGTGT‑CCC‑TATCTGTTACAGTCTCCTAAAGTATCCTCCTAAAGTCACCTCCTAACGTCCATCCTAAAGCCAACACCTAAAGCCTACACCTAAAGACCCATCAAGTCAACGCCTATCTTAAAGTTTAAACATAAAGACCAGACCTAAAGACCAGACCTAAAGACACTACATAAAGACCAGACCTAAAGACGCCTTGTTGTTAGCCATAAAGTGATAACCTTTAATCATTg                                                                                                                                                                                                            <  1:250455/301‑1 (MQ=255)`  
                                                                                                     `gggTT‑CCCTAATGGTTGGGGATGACCCTTGGGTTTGTCTTTGGGTGTTACCTTGAGTGTCTCTCTGTGT‑CCC‑TATCTGTTACAGTCTCCTAAAGTATCCTCCTAAAGTCACCTCCTAACGTCCATCCTAAAGCCAACACCTAAAGCCTACACCTAAAGACCCATCAAGTCAACGCCTATCTTAAAGTTTAAACATAAAGACCAGACCTAAAGACCAGACCTAAAGACACTACATAAAGACCAGACCTAAAGACGCCTTGTTGTTAGCCATAAAGTGATAACCTTTAATCATTGTCTttatt                                                                                                                                                                                                    <  1:358006/301‑1 (MQ=255)`  
                                                                                                           `cccTAAGGGTTGGGGATGACCCTTGGGTTTGTCTTTGGGTGTTACCTTGAGTGTCTCTCTGTGT‑CCC‑TATCTGTTACAGTCTCCTAAAGTATCCTCCTAAAGTCACCTCCTAACGTCCATCCTAAAGCCAACACCTAAAGCCTACACCTAAAGACCCATCAAGTCAACGCCTATCTTAAAGTTTAAACATAAAGACCAGACCTAAAGACCAGACCTAAAGACACTACATAAAGACCAGACCTAAAGACGCCTTGTTGTTATCCATAAAGTGATAACCTTTAATCATTGTCTTTATTAATAc                                                                                                                                                                                               <  1:409085/301‑1 (MQ=255)`  
                                                                                                           `cccTAAGGGTTGGGGATGACCCTTGGGTTTGTCTTTGGGTGTTACCTTGAGTGTCTCTCTGTGT‑CCC‑GATCTGTTACAGTCTCCTAAAGTATCCTCCTAAAGTCACCTCCTAACGTCCATCCTAAAGCCAACACCTAAAGCCTACACCTAAATACCCATCAAGTCAACGCCTATCTTAAAGTTTAAACATAAAGACCAGACCTAAAGACCAGACCTAAAGACACTACATAAAGACCAGACCTAAAGACGCCTTGTTGTTAGCCATAAAGTGATAACCTTTAATCATTGTCTTTATTAATAc                                                                                                                                                                                               <  1:31318/301‑1 (MQ=255)`  
                                                                                                           `agatgtgtagaagtgacagcccTTGGGTTTGTCTTTGGGTGTTACCTTGAGTGTCTCTCTGTGT‑CCC‑GATCTGTTACAGTCTCCTAAAGTATCCTCCTAAAGTCACCTCCTAACGTCCATCCTAAAGCCAACACCTAAAGCCTACACCTAAAGACCCATCAAGTCAACGCCTATCTTAAAGTTTAAACATAAAGACCAGACCTAAAGACCAGACCTAAAGACACTACATAAAGACCAGACCTAAAGACGCCTTGTTGTTAGCCATAAAGTGATAACCTTTAATCATTGTCTTTATTAATAc                                                                                                                                                                                               <  1:306242/282‑1 (MQ=255)`  
                                                                                                            `ccTAAGGGTTGGGGATGACCCTTGGTTTTGTCTTTGGGTGTTACCTTGAGTGTCTCTCTGTGT‑CCCTTATCTGTTCCAGTCTCCTAAAGTATCCTCCTAAAGTCACCTCCTAACGTCCATCCTAAAGCCAACACCTAAAGCCTACACCTAAAGACCCATCAAGTCAACGCCTATCTTAAAGTTTAAACATAAAGACCAGACCTAAAGACCAGACCTAAAGACACTACATAAAGACCAGACCTAAAGACGCCTTGTTGTTAGCCATAAAGTGATAACCTTTAATCATTGTCTTTATTAATAc                                                                                                                                                                                               <  1:271898/301‑1 (MQ=255)`  
                                                                                                                 `gggTTGTGGATGACCCTTGGGTTTGTCTTTGGGTGTTACCTTGAGTGTCTCTCTGTGT‑CCC‑TATCTGTTACAGTCTCCTAAAGTATCCTCCTAAAGTCACCTCCTAACGTCCATCCTAAAGCCAACACCTAAAGCCTACACCTAAAGACCCATCAAGTCAACGCCTATCTTAAAGTTTAAACATAAAGACCAGACCTAAAGACCAGACCTAAAGACACTACATAAAGACCAGACCTAAAGACGCCTTGTTGTTAGCCATAAAGTGATAACCTTTAATCATTGTCTTTATTAATACAACTCa                                                                                                                                                                                         >  1:663516/1‑301 (MQ=255)`  
                                                                                                                 `gggTTGGGGATGACCCTTTGGTTTGTCTTTGGGAGTTACCTTGAGTGTCTCTCTGTGT‑CCC‑GATCTGTTACAGTCTCCTAAAGTATCCTCCTAAAGTCACCTCCTAACGTCCATCCTAAAGCCAACACCTAAAGCCTACACCTAAAGACCCATCAAGTCAACGCCTATCTTAAAGTTTAAACATAAAGACCAGACCTAAAGACCAGACCTAAAGACACTACATAAAGACCAGACCTAAAGACGCCTTGTTGTTAGCCATAAAGTGATAACCTTTAATcctgtctcttatacacatctccga                                                                                                                                                                                         >  1:550092/1‑278 (MQ=255)`  
                                                                                                                 `gggTTGGGGATGACCCTTGGGTTTGTCTTTGGGTGTTACCTTGAGTGTCTCTCTGTGT‑CCC‑TATCTGTTACAGTCTCCTAAAGTATCCTCCTAAAGTCACCTCCTAACGTCCATCCTAAAGCCAACACCTAAAGCCTACACCTAAAGACCCATCAAGTCAACGCCTATCTTAAAGTTTAAACATAAAGACCAGACCTAAAGACCAGACCTACAGACACTACATAAAGACCAGACCTAAAGACGCCTTGTTGTTAGCCATAAAGTGATAACCTTTAATCATTGTCTTTATTCATACAAATCa                                                                                                                                                                                         >  1:62738/1‑301 (MQ=255)`  
                                                                                                                     `tGGGTATGACCCTTGGGTTTGTCTTTGGGTGTTACCCTGAGTGTCTCTCTGTGT‑CCT‑GATCTGTTACAGTCTCCTAAAGTATCCTCCTAAAGTCACCTCCTAACGTCCATCCTAAAGCCAACACCTAAAGCCTACACCTAAAGACCCATCAAGTCAACGCCTATCTTAAAGTTTAAACATAAAGACCAGACCTAAAGACCAGACCTAAAGACACTACATAAAGACCAGACCTAAAGACGCCTTGTTGTTAGCCATAAAGTGATAACCTTTAATCATTGTCTTTATTAATACAACTCACtat                                                                                                                                                                                     <  1:264189/301‑1 (MQ=255)`  
                                                                                                                        `ggATGACCCTTGGGTTTGTCTTTGGGTGTTACCTTGAGTGTCTCTCTGTGT‑CCC‑TATCTGTTACAGTCTCCTAAAGTATCCTCCTAAAGTCACCTCCTAACGTCCATCCTAAAGCCAACACCTAAAGCCTACACCTAAAGACCCATCAAGTCAACGCCTATCTTAAAGTTTAAACATAAAGACCAGACCTAAAGACCAGACCTAAAGACACTACATAAAGACCAGACCTAAAGACGCCTTGTTGTTAGCCATAAAGTGATAACCTTTAATCATTGTCTTTATTAATACAACTCACTATAAg                                                                                                                                                                                  <  1:710052/301‑1 (MQ=255)`  
                                                                                                                         `gATGACCCTTGGGTTTGTCTTTGGGTGTTACCTTGAGTGTCTCTCTGTGT‑CCC‑TATCTGTTACAGTCTCCTAAAGTATCCTCCTAAAGTCACCTCCTAACGTCCATCCTAAAGCCAACACCTAAAGCCTACACCTAAAGACCCATCAAGTCAACGCCTATCTTAAAGTTTAAACATAAAGACCAGACCTAAAGACCAGACCTAAAGACACTACATAAAGACCAGACCTAAAGACGCCTTGTTGTTAGCCATAAAGTGATAACCTTTAATCATTGTCTTTATTAATACAACTCACTATAAgg                                                                                                                                                                                 <  1:234732/301‑1 (MQ=255)`  
                                                                                                                          `accgcccGTGGGTTTGGCTTTGGGTGTTACCTTGAGTGTTGCTCTGTGT‑CCC‑TATCTGTTACAGTCTCCTAAAGTATCCTCCTAAAGTCACCTCCTAACGTCCATCCTAAAGCCAACACCTAAAGCCTACACCTAAAGACCCATCAAGTCAACGCCTATCTTAAAGTTTAAACATAAAGACCAGACCTAAAGACCAGACCTAAAGACACTACATAAAGACCAGACCTAAAGACGCCTTGTTGTTAGCCATAAAGTGATAACCTTTAATCATTGTCTTTATTAATACAACTCACTATAAGga                                                                                                                                                                                <  1:45168/297‑1 (MQ=255)`  
                                                                                                                          `aTGACCCTTGGGTTTGTCTTTGGGTGTTACCTTGAGTGTCTCTCTGTGT‑CCC‑TATCTGTTCCAGTCTCCTAAAGTATCCTCCTAAAGTCACCTCCTAACGTCCATCCTAAAGCCAACACCTAAAGCCTACACCTAAAGACCCATCAAGTCAACGCCTATCTTAAAGTTTAAACATAAAGACCAGACCTAAAGACCAGACCTAAAGACACTACATAAAGACCAGACCTAAAGACGCCTTGTTGTTAGCCATAAAGTGATAACCTTTAATCATTGTCTTTATTAATACAACTCACTATAAGga                                                                                                                                                                                <  1:694443/301‑1 (MQ=255)`  
                                                                                                                              `cccTTGGGTTTGTCTTTGGGTGTTACCTTGAGTGTCTCTCTGTGT‑CCC‑TATCTGTTACAGTCTCCTAAAGTATCCTCCTAAAGTCACCTCCTAACGTCCATCCTAAAGCCAACACCTAAAGCCTACACCTAAAGACCCATCAAGTCAACGCCTATCTTAAAGTTTAAACATAAAGACCAGACCTAAAGACCAGACCTAAAGACACTACATAAAGACCAGACCTAAAGACGCCTTGTTGTTAGCCATAAAGTGATAACCTTTAATCATTGTCTTTATTAATACAACTCACTATAAGgagaga                                                                                                                                                                            >  1:386286/1‑301 (MQ=255)`  
                                                                                                                              `cccTTGGGTTTGTCTTTGGGTGTTACCTTGAGTGTCTCTCTGTGT‑CCC‑GATCTGTTACAGTCTCCTAAAGTATCCTCCTAAAGTCACCTCCTAACGTCCATCCT‑‑AGCCAACACCTAAAGCCTACACCTAAAGACCCATCAAGTAAACGCCTATCTTAAAGTTTAAACATAAAGACCAGACCTAAAGACCAGACCTAAAGACACTACATAAAGACCAGACCTAAAGACGCCTTGTTGTTAGCCATAAAGTGATAACCTTTAATCATTGTCTTTATTAATACAACTCACTATAAGGAGAGACa                                                                                                                                                                          <  1:206846/301‑1 (MQ=255)`  
                                                                                                                                   `gggTTTGTCTTTGGGTGTTACGTTGAGTGTCTCTCTGTGT‑CCC‑GATCTGTAACAGTCTCCTAAAGTATCCTCCTAAAGTCACCTCCTAACGTCCATCCTAAAGCCAACACCTAAAGCCTACACCTAAAGACCCATCAAGTCAACGCCTATCTTAAAGTTTAAACATAAAGACCAGACCTAAAGACCAGACCTAAAGACACTACATAAAGACCAGACCTAAAGACGCCTTGTTGTTAGCCATAAAGTGATAACCGTTAATCATTGTCTTTATTAATACAACTCACTATAAGGAGAGACAACt                                                                                                                                                                       <  1:732460/301‑1 (MQ=255)`  
                                                                                                                                         `gTCTTTGGGTGTTACCTTGAGTGTCTCTCTGTGTCCCC‑TATCTGTTACAGTCTCCTAAAGTATCCTCCTAAAGTCACCTCCTAACGTCCATCCTAAAGCCAACACCTAAAGCCTACACCTAAAGACCCATCAAGTCAACGCCTATCTTAAAGTTTAAACATAAAGACCAGACCTAAAGACCAGACCTAAAGACACTACATAAAGACCAGACCTAAAGACGCCTTGTTGTTAGCCATAAAGTGATAACCTTTAATCATTGTCTTTATTAATACAACTCACTATAAGGAGAGACAACTTAAag                                                                                                                                                                  >  1:146664/1‑301 (MQ=255)`  
                                                                                                                                         `gTCTTTGGGTGTTACCTTGAGTGTCTCTCTGTGT‑CCC‑TATCTGTTACAGTCTCCTAAAGTATCCTCCTAAAGTCACCTCCTAACGTCCATCCTAAAGCCAACACCTAAAGCCTACACCTAAAGACCCATCAAGTCAACGCCTATCTTAAAGTTTAAACATAAAGACCAGACCTAAAGACCAGACCTAAAGACACTACATAAAGACCAGACCTAAAGACGCCTTGTTGTTAGCCATAACGTGATAACCTTTAATCATTGTCTTTATTAATACAACTCACTATAAGGAGAGACAACTTAAaga                                                                                                                                                                 >  1:678046/1‑301 (MQ=255)`  
                                                                                                                                         `gTCTTTGGGTGTTACCTTGAGTGTCTCTCTGTGT‑CCC‑GATCTGTTACAGTCTCCTAAAGTATCCTCCTAAAGTCACCTCCTAACGTCCATCCTAAAGCCAACACCTAAAGCCTACACCTAAAGACCCATCAAGTCAACGCCTATCTTAAAGTTTAAACATAAAGACCAGACCTAAAGACCAGACCTAAAGACACTACATAAAGACCAGACCTAAAGACGCCTTGTTGTTAGCCATAAAGTGATAACCTTTAATCATTGTCTTTATTAATACAACTCACTATAAGGAGAGACAACTTAAaga                                                                                                                                                                 >  1:292420/1‑301 (MQ=255)`  
                                                                                                                                          `aagagacaggtTACCTTGAGTGTCTCTCTGTGT‑CCC‑GATCTGTTACAGTCTCCTAAAGTATCCTCCTAAAGTCACCTCCTAACGTCCATCCTAAAGCCAACACCTAAAGCCTACACCTAAAGACCCATCAAGTCAACGCCTATCTTAAAGTTTAAACATAAAGACCAGACCTAAAGACCAGACCTAAAGACACTACATAAAGACCAGACCTAAAGACGCCTTGTTGTTAGCCATAAAGTGATAACCTTTAATCATTGTCTTTATTAATACAACTCACTATAAGGAGAGACAACTTAAagag                                                                                                                                                                <  1:61277/292‑1 (MQ=255)`  
                                                                                                                                                      `aCCTTGAGTGTCTCTCTGTGT‑CCC‑TATCTGTTACAGTCTCCTAAAGTATCCTCCTAAAGTCACCTCCTAACGTCCATCCTAAAGCCAACACCTAAAGCCTACACCTAAAGACCCATCAAGTCAACGCCTATCTTAAAGTTTAAACATAAAGACCAGACCTAAAGACCAGACCTAAAGACACTACATAAAGACCAGACCTAAAGACGCCTTGTTGTTAGCCATAAAGTGATAACCTTTAATCATTGTCTTTATTAATACAACTCACTATAAGgactgtctcttatacacatctcagagccca                                                                                                                                                    >  1:130995/1‑273 (MQ=255)`  
                                                                                                                                                                 `ctctctGTGT‑CCC‑TATCTGTTACAGTCTCCTAAAGTATCCTCCTAAAGTCACCTCCTAACGTCCATCCTAAAGCCAACACCTAAAGCCTACACCTAAAGACCCATCAAGTCAACGCCTATCTTAAAGTTTAAACATAAAGACCAGACCTAAAGACCAGACCTAAAGACACTACATAAAGACCAGACCTAAAGACGCCTTGTTGTTAGCCATAAAGTGATAACCTTTAATCATTGTCTTTATTAATACAACTCACTATAAGGAGAGACAACTTAAagagctgtctcttatacacatctccga                                                                                                                                         >  1:284596/1‑278 (MQ=255)`  
                                                                                                                                                                                 `aTCTGTTACAGTCTCCTAAAGTATCCTCCTAAAGTCACCTCCTAACGTCCATCCTAAAGCCAACACCTAAAGCCTACACCTAAAGACCCATCAAGTCAACGCCTATCTTAAAGTTTAAACATAAAGACCAGACCTAAAGACCAGACCTAAAGACACTACATAAAGGCCAGACCTAAAGACGCCTTGTTGTTAGCCATAAAGTGATAACCTTTAATCATTGTCTTTATTAATACAACTCACTATAAGGAGAGACAACTTAAAGAGACTTAAAAGATTAATTTAAAATTTATCAAAAAGAGTa                                                                                                                           >  1:804117/1‑301 (MQ=255)`  
                                                                                                                                                                                 `aTCTGTTACAGTCTCCTAAAGTATCCTCCTAAAGTCACCTCCTAACGTCCATCCTAAAGCCAACACCTAAAGCCTACACCTAAAGACCCATCAAGTCAACGCCTATCTTAAAGTTTAAACATAAAGACCAGACCTAAAGACCAGACCTAAAGACACTACATAAAGACCAGACCTAAAGACGCCTTGTTGTTAGCCATAAAGTGATAACCTTTAATCATTGTCTTTATTAATACAACTCACTATAAGGAGAGACAACTTAAAGAGACTTACAAGATTCATTTAAAATTTATCAAAAAGAGTa                                                                                                                           >  1:765791/1‑301 (MQ=255)`  
                                                                                                                                                                                 `aTCTGTTACAGTCTCCTAAAGTATCCTCCTAAAGTCACCTCCTAACGTCCATCCTAAAGCCAACACCTAAAGCCTACACCTAAAGACCCATCAAGTCAACGCCTATCTTAAAGTTTAAACAAAAAGACCAGACCTAAAGACCAGACCTAAAGACACTACATAAAGACCAGACCTAAAGACGCCTTGTTGTTAGCCATAAAGTGATAACCTTTAATCATTGTCTTTATTAATACAACTCACTATAAGGAGAGACAACTTAAAGAGACTTAAAAGATTAATTTAAAATTTATCAAAAAGAGTa                                                                                                                           >  1:574585/1‑301 (MQ=255)`  
                                                                                                                                                                                   `cTGTTACAGTCTCCTAAAGTATCCTCCTAAAGTCACCTCCTAACGTCCATCCTAAAGCCAACACCTAAAGCCTACACCTAAAGACCCATCAAGTCAACGCCTATCTTAAAGTTTAAACATAAAGACCAGACCTAAAGACCAGACCTAAAGACACTACATAAAGACCAGACCTAAAGACGCCTTGTTGTTAGCCATAAAGTGATAACCTTTAATCATTGTCTTTATTAATACAACTCACTATAAGGAGAGACAACTTAAAGAGACTTAAAAGATTAATTTAAAATTTATAAAAAAGAGTAtt                                                                                                                         >  1:115602/1‑301 (MQ=255)`  
                                                                                                                                                                                    `tGTTACAGTCTCCTAAAGTATCCTCCTAAAGTCACCTCCTAACGTCCATCCTAAAGCCAACACCTAAAGCCTACACCTAAAGACCCATCAAGTCAACGCCTATCTTAAAGTTTAAACATAAAGACCAGACCTAAAGACCAGACCTAAAGACACTACATAAAGACCAGACCTAAAGACGCCTTGTTGTTAGCCATAAAGTGATAACCTTTAATCATTGTCTTTATTAATACAACTCACTATAAGGAGAGACAACTTAAAGAGACTTAAAAGATTAATTTAAAATTTATCAAAAAGAGTATTg                                                                                                                        >  1:93134/1‑301 (MQ=255)`  
                                                                                                                                                                                      `ttACAGTCTCCTAAAGTATCCTCCTAAAGTCACCTCCTAACGTCCATCCTAAAGCCAACACCTAAAGCCTACACCTAAAGACCCATCAAGTCAACGCCTATCTTAAAGTTTAAACATAAAGACCAGACCTAAAGACCAGACATAAAGACACTACATAAAGACCAGACCTAAAGACGCCTTGTTGTTAGCCATAAAGTGATAACCTTTAATCATTGTCTTTATTAATACAACTCACTATAAGGAGAGACAACTTAAAGAGACTTAAAAGATTAATTTAAAATTTATCAAAAAGAGTATTGAc                                                                                                                      <  1:193832/301‑1 (MQ=255)`  
                                                                                                                                                                                       `tACAGTCTCCTAAAGTATCCTCCTAAAGTCACCTCCTAACGTCCATCATAAAGCCAACACCTAAAGACTACACCTAAAGACCCATCAAGGCAACTCCTATCTTAAAGTTTAAACATAAAGACCAGACCTAAAGTCCAGACCTAAAGACACTACATAAAGACCAGACCTAAAGACGCCTTGTTGTTAGCCATAAAGTGATAACCTTTAATCATTGTCTTTATTAATACAACTCACTATAAGGAGAGACAACTTAAAGAGACTTAAAAGATTAATTTAAAATTTATCAAAAAGAGTATTGACt                                                                                                                     <  1:658302/301‑1 (MQ=255)`  
                                                                                                                                                                                            `tgtataagagacagctcctAAAGTCACCTCCTAACGTCCATCCTAAAGCCAACACCTAAAGCCTACACCTAAAGACCCATCAAGTCAACGCCTATCTTAAAGTTTAAACATAAAGACCAGACCTAAAGACCAGACCTAAAGACACTACATAAAGACCAGACCTAAAGACGCCTTGTTGTTAGCCATAAAGTGATAACCTTTAATCATTGTCTTTATTAATACAACTCACTATAAGGAGAGACAACTTAAAGAGACTTAAAAGATTAATTTAAAATTTATCAAAAAGAGTATTGACTTAAAg                                                                                                                <  1:782236/287‑1 (MQ=255)`  
                                                                                                                                                                                             `ctcCTAAAGTATCCTCCTAAAGTCACCTCCTAACGTCCATCCTAAAGCCAACACCTAAAGCCTACACCTAAAGACCCATCAAGTCAACGCCTATCTTAAAGTTTAAACATAAAGACCAGACCTAAAGACCAGACCTAAAGACACTACATAAAGACCAGACCTAAAGACGCCTTGTTGTTAGCCATAAAGTGATAACCTTTAATCATTGTCTTTATTAATACAACTCACTATAAGGAGAGACAACTTAAAGAGACTTAAAAGATTAATTTAAAATTTATCAAAAAGAGTATTGACTTAAAGt                                                                                                               >  1:227715/1‑301 (MQ=255)`  
                                                                                                                                                                                               `ccTAAAGTATCCTCCTAAAGTCACCTCCTAACGTCCATCCTAAAGCCAACACCTAAAGCCTACACCTAAAGACCCATAAAGTCAACGCCTATCTTAAAGTTTAAACATAAAGACCAGACCTAAAGACCAGACCTAAAGACACTACATAAAGACCAGACCTAAAGACGCCTTGTTGTTAGCCATAAAGTGATAACCTTTAATCATTGTCTTTATTAATACAACTCACTATAAGGAGAGACAACTTAAAGAGACTTAAAAGATTAATTTAAAATTTATCAAAAAGAGTATTGACTTAAAgctt                                                                                                             >  1:155769/1‑298 (MQ=255)`  
                                                                                                                                                                                                             `ctAAAGTCACCTCCTAACGTCCATCCTAAAGCCAACACCTAAAGCCTACACCTAAAGACCCATCAAGTCAACGCCTATCTTAAAGTTTAAACATAAAGACCAGACCTAAAGACCAGACCTAAAGACACTACATAAAGACCAGACCTAAAGACGCCTTGTTGTTAGCCATAAAGTGATAACCTTTAATCATTGTCTTTATTAATACAACTCACTATAAGGAGAGACAACTTAAAGAGACTTAAAAGATTAATTTAAAATTTATCAAAAAGAGTATTGACTTAAAGTCTAACCTATAGGATAc                                                                                               <  1:402420/301‑1 (MQ=255)`  
                                                                                                                                                                                                             `ctAAAGTCACCTCCTAACGTCCATCCTAAAGCCAACAACTAAAGCCTACACCTAAAGACCCATCAAGTCAACGCCTATCTTAAAGTTTAAACATAAAGACCAGACCTAAAGACCAGACCTAAAGACAATACATAAAGACCAGACCTAAAGACGCCTTGTTGTTAGCCATAAAGTGATAACCTTTAATCATTGTCTTTATTAATACAACTCACTATAAGGAGAGACAACTTAAAGAGACTTAAAAGATTAATTTAAAATTTATCAAAAAGAGTATTGACTTAAAGTCTAACCTATAGGATAc                                                                                               <  1:490234/301‑1 (MQ=255)`  
                                                                                                                                                                                                             `ctAAAGTCACCTCCTAACGGACATCCTAAAGCCAACACCTAAAGCCTACACCTAAAGACCCATCAAGTCAACGCCTATCTTAAAGTTTAAACATAAATACCAGACCTAAAGACCAGACCTAAAGACACTACATAAAGACCAGACCTAAAGACGCCTTGTTGTTAGCCATAAAGTGATAACCTTTAATCATTGTCTTTATTAATACAACTCACTATAAGGAGAGACAACTTAAATAGACTTAAAAGATTAATTTAAAATTTATCAAAAATAGTATTGACTTAAAGTCTAACCTATAGGATAc                                                                                               <  1:790445/301‑1 (MQ=255)`  
                                                                                                                                                                                                             `cggagatgggtataagagacagaTCCTAAAGCCAACACCTAAAGCCTACACCTAAAGACCCATCAAGTCAACGCCTATCTTAAAGTTTAAACATAAAGACCAGACCTAAAGACCAGACCTAAAGACACTACATAAAGACCAGACCTAAAGACGCCTTGTTGTTAGCCATAAAGTGATAACCTTTAATCATTGTCTTTATTAATACAACTCACTATAAGGAGAGACAACTTAAAGAGACTTAAAAGATTAATTTAAAATTTATCAAAAAGAGTATTGACTTAAAGTCTAACCTATAGGATAc                                                                                               <  1:221302/279‑1 (MQ=255)`  
                                                                                                                                                                                                                 `aGTCACCTACCAACGTCCAACCGAAAGACAACACCTAAAGCCTAAACCTGAAGACCCATAAAGTCAACGCCTATCTTAAAGTTTAAACAGAAAGACCAGACCTAAAGACCAGACCTAAAGACACTACATAAAGACCAGACCTAAAGACGCCTTGTTGTGAGCCATAAAGTGATAACCTTTAATCATTGTCTTTATTAATACAACTCACTATAAGGAGAGACAACTTAAAGAGACTTAAAAGATTAATTTAAAATTTATCAAAAAGAGTATGGACTTAAAGTCTAACCTATAGGATACTTAc                                                                                           <  1:79729/301‑1 (MQ=255)`  
                                                                                                                                                                                                                    `tgggctcggagaagtgtataagagacagcacCTAAAGCCTACACCGAAAGACCCATCAAGTCAACGCCTATCGTAAAGTTTAAACATAAAGACCAGACCTAAAGACCAGACCTAAAGACACTACATAAAGAACAGACCTAAAGACGCCTTGTTGTTAGCCATAAAGTGATAACCTTTAATCATTGTCTTTATTAATACAACTCACTATAAGGAGAGACAACTTAAAGAGACTTAAAAGATTAATTTAAAATTTATCAAAAAGAGTATTGACTTAAAGTCTAACCTATAGGATACTTACAGc                                                                                        <  1:416438/273‑1 (MQ=255)`  
                                                                                                                                                                                                                     `acccactAGCGTCCATCCTAAAGCCAACACCTAAAGCCTACACCTAAAGACCCATCAAGTCAACGCCTATCTTAAAGTTTAAACATAAAGACCAGACCTAAAGACCAGACCTAAAGACACTACATAAAGACCAGACCTAAAGACGCCTTGTTGTTAGCCATAAAGTGATAACCTGTAATCATTGTCTTTATTAATACAACTCACTATAAGGAGAGACAACTTAAAGAGACTTAAAAGATTAATTTAAAATTTATCAAAAAGAGTATTGACTTAAAGTCTAACCTATAGGATACTTACAGcc                                                                                       <  1:364831/296‑1 (MQ=255)`  
                                                                                                                                                                                                                     `aCCTCCTAACGTCCATCCTAAAGCCAACACCTAAAGCCTACACCTAAAGACCCATCAAGTCAACGCCTATCTTAAAGTTTAAACATAAAGACCAGACCTAAAGACCAGACCTAAAGACACTACATAAAGACCAGACCTAAAGACGCCTTGTTGTTAGCCATAAAGTGATAACCTTTAATCATTGTCTTTATTAATACAACTCACTATAAGGAGAGACAACTTAAAGAGACTTAAAAGATTAATTTAAAATTTATCAAAAAGAGTATTGACTTAAAGTCTAACCTATAGGATACTTACAGcc                                                                                       <  1:342689/301‑1 (MQ=255)`  
                                                                                                                                                                                                                                    `tCCTAAAGCCAACACCTAAAGCCTACACCTAAAGACCCATCAAGTCAACGCCTATCTTAAAGTTTAAACATAAAGACCAGACCTAAAGACCAGACCTAAAGACACTACATAAAGACCAGACCTAAAGACGCCTTGTTGTTAGCCATAAAGTGATAACCTTTAATCATTGTCTTTATTAATACAACTCACTATAAGGAGAGACAACTTAAAGAGACTTAAAAGATTAATTTAAAATTTATCAAAAAGAGTATTGACTTAAAGTCTAACCTATAGGATACTTACAGccctgtctcttatacac                                                                        >  1:474471/1‑286 (MQ=255)`  
                                                                                                                                                                                                                                    `tCCTAAAGCCAACACCTAAAGCCTACACCTAAAGACCCATCAAGTCAACGCCTATCTTAAAGTTTAAACATAAAGACCAGACCTAAAGACCAGACCTAAAGACACTACATAAAGACCAGACCTAAAGACGCCTTGTTGTTAGCCATAAAGTGATAACCTTTAATCATTGTCTTTATTAATACAACTCACTATAAGGAGAGACAACTTAAAGAGACTTAAAAGATTAATTTAAAATTTATCAAAAAGAGTATTGACTTAAAGTCTAACCTATAGGATACTTACAGCCATCGAGAGGGACACg                                                                        >  1:371722/1‑301 (MQ=255)`  
                                                                                                                                                                                                                                    `tCCTAAAGCCAACACCTAAAGCCTACACCTAAAGACCCATCAAGTCAACGCCTATCTTAAAGTTTAAACATAAAGACCAGACCTAAAGACCAGACCTAAAGACACTACATAAAGACCAGACCTAAAGACGCCTTGTTGTTAGCCATAAAGTGATAACCTTTAATCATTGTCTTTATTAATACAACTCACTATAAGGAGAGACAACTTAAAGAGACTTAAAAGATTAATTTAAAATTTAACAAAAAGAGTATTGACTTAAAGTCTAACCTATAGGATACTTACAGCCTTCGAGAGGGACACg                                                                        >  1:597332/1‑301 (MQ=255)`  
                                                                                                                                                                                                                                             `cAACACCTAAAGCCTACACCTAAAGACCCATCAAGTCAACGCCTATCTTAAAGTTTAAACATAAAGACCAGACCTAAAGACCAGACCTAAAGACACTACATAAAGACCAGACCTAAAGACGCCTTGTTGTTAGCCATAAAGTGATAACCTTTAATCATTGTCTTTATTAATACAACTCACTATAAGGAGAGACAACTTAAAGATACTTAAAAGATTAATTTAATATTTATCAAAAAGAGTATTGACTTAAAGTCTAACCTATAGGATACTTACAGCCATCGAGAGGGACACGGCGAATAGc                                                               <  1:825619/301‑1 (MQ=255)`  
                                                                                                                                                                                                                                              `aacaacTAAAGCCTACACCTAAAGACCCATCAAGTCAACGCCTATCTTAAAGTTTAAACATAAAGACCAGACCTAAAGACCAGACCTAAAGACACTACATAAAGACCAGACCTAAAGACGCCTTGTTGTTAGCCATAAAGTGATAACCTTTAATCATTGTCTTTATTAATACAACTCACTATAAGGAGAGACAACTTAAAGAGACTTAAAAGATTAATTTAAAATTTATCAAAAAGAGTATTGACTTAAAGTCTAACCTATAGGATACTTACAGCCATCGAGAGGGACACGGCGAATAGcc                                                              <  1:138419/301‑1 (MQ=255)`  
                                                                                                                                                                                                                                              `aaCACCTAAAGCCTACACCTAAAGACCCATCAAGTCAACGCCTATCTTAAAGTTTAAACATAAAGACCAGACCTAAAGACCAGACCTAAAGACACTACATAAAGACCAGACCTAAAGACGCCTTGTTGTTAGCCATAAAGTGATAACCTTTAATCATTGTCTTTATTAATACAACTCACTATAAGGAGAGACAACTTAAAGAGACTTAAAAGATTAATTTAAAATTTATCAAAAAGAGTATTGACTTAAAGTCTAACCTATAGGATACTTACAGCCATCGAGAGGGACACGGCGAATAGcc                                                              <  1:671064/301‑1 (MQ=255)`  
                                                                                                                                                                                                                                               `acacCTAAAGCCTACACCTAAAGACCCATCAAGTCAACGCCTATCTTAAAGTTTAAACATAAAGACCAGACCTAAAGACCAGACCTAAAGACACTACATAAAGACCAGACCTAAAGACGCCTTGTTGTTAGCCATAAAGTGATAACCTTTAATCATTGTCTTTATTAATACAACTCACTATAAGGAGAGACAACTTAAAGAGACTTAAAAGATTAATTTAAAATTTATCAAAAAGAGTATTGACTTAAAGTCTAACCTATAGGATACTTACAGccctgtctcttatacacatctccgagcc                                                             >  1:512672/1‑275 (MQ=255)`  
                                                                                                                                                                                                                                                  `ccTAAAGCCTACACCTAAAGACCCATCAAGTCAACGCCTATCTTAAAGTTTAAACATAGAGACCAGACCTAAAGACCAGACCTAAAGACACTACATAAAGACCAGACCTAAAGACGCCTTGTTGTTAGCCATAAAGTGATAACCTTTAATCATTGTCTTTATTAATACAACTCACTATAAGGAGAGACAACTTAAAGAGACTTAAAAGATTAATTTAAAATTTATCAAAAAGAGTATTGACTTAAAGTCTAACCTATAGGATACTTACAGCCATCGAGAGGGACACGGCGAATAGCCATcc                                                          >  1:256872/1‑301 (MQ=255)`  
                                                                                                                                                                                                                                                  `ccTAAAGCCTACACCTAAAGACCCATCAAGTCAACGCCTATCTTAAAGTTTAAACATAAAGACCAGACCTAAAGACCAGACCTAAAGACACGACATAAAGACCAGACCTAAAGACGCCTTGTTGTTAGCCATAAAGTGATAACCTTTAATCATTGTCTTTATTAATACAACTCACTATAAGGAGAGACAACTTAAAGAGACTTAAAAGATTAATTTAAAATTTATCAAAAAGAGTATTGACTTAAAGTCTAACCTATAGGATACTTACAGACATCGATAGGGACACGGCGAATAGCCATcc                                                          >  1:106876/1‑301 (MQ=255)`  
                                                                                                                                                                                                                                                        `gCCTACACCTAAAGACCCATCAAGTCAACGCCTATCTTAAAGTTTAAACATAAAGACCAGACCTAAAGACCAGACCTAAAGACACTACATAAAGACCAGACCTAAAGACGCCTTGTTGTTAGCCATAAAGTGATAACCTTTAATCATTGTCTTTATTAATACAACTCACTATAAGGAGAGACAACTTAAAGAGACTTAAAAGATTAATTTAAAATTTATCAAAAAGAGTATTGACTTAAAGTCTAACCTATAGGATACTTACAGCCATCGAGAGGGACACGGCGAATAGCCATCCCAATCg                                                    >  1:84652/1‑301 (MQ=255)`  
                                                                                                                                                                                                                                                               `ccTAAAGACCCATCAAGTCAACGCCTATCTTAAAGTTTAAACATAAAGACCAGACCTAAAGACCAGACCTAAAGACACTACATAAAGACCAGACCTAAAGACGCCTTGTTGTTAGCCATAAAGTGATAACCTTTAATCATTGTCTTTATTAATACAACTCACTATAATGAGAGACAACTTAAAGAGACTTAAAAGATTAATTTAAAATTTATCAAAAAGAGTATTGACTTAAAGTCTAACCTATAGTATACTTACAGCCATCGAGAGGGACACGGCGAATAGCCATCCCAATCGACACCgg                                             >  1:177817/1‑301 (MQ=255)`  
                                                                                                                                                                                                                                                               `ccTAAAGACCCATCAAGTCAACGCCTATCTTAAAGTTTAAACATAAAGACCAGACCTAAAGACCAGACCTAAAGACACTACATAAAGACCAGACCTAAAGACGCCTTGTTGTTAGCCATAAAGTGATAACCTTTAATCATTGTCTTTATTAATACAACTCACTATAAGGAGAGACAACTTAAAGAGACTTAAAAGATTAATTTAAAATTTATCAAAAAGAGTATTGACTTAAAGTCTAACCTATAGGATACTTACAGCCATCGAGAGGGACACGGCGAATAGCCATCCCAATCGACACCgg                                             >  1:54393/1‑301 (MQ=255)`  
                                                                                                                                                                                                                                                                      `aCCCATCAAGTCAACGCCTATCTTAAAGTTTAAACATAAAGACCAGACCTAAAGACCAGACCTAAAGACACTACATAAAGACCAGACCTAAAGACGCCTTGTTGTTAGCCATAAAGTGATAACCTTTAATCATTGTCTTTATTAATACAACTCACTATAAGGAGAGACAACTTAAAGAGACTTAAAAGATTAATTTAAAATTTATCAAAAAGAGTATTGACTTAAAGTCTAACCTATAGGATACTTACAGCCATCGAGAGGGACACGGCGAATAGCCATCCCAATCGACACCGGGGTCAAc                                      <  1:529633/301‑1 (MQ=255)`  
                                                                                                                                                                                                                                                                              `aGTCAACGCCTATCTTAAAGTTTAAACATAAAGACCAGACCTAAAGACCAGACCTAAAGACACTACATAAAGACCAGACCTAAAGACGCCTTGTTGTTAGCCATAAAGTGATAACCTTTAATCATTGTCTTTATTAATACAACTCACTATAAGGAGAGACAACTTAAAGAGACTTAAAAGATTAATTTAAAATTTATCAAAAAGAGTATTGACTTAAAGTCTAACCTATAGGATACTTACAGCCATCGAGAGGGACACGGCGAATAGCCATCCCAATCGACACCGGGGTCAACCGGATAAg                              >  1:717721/1‑301 (MQ=255)`  
                                                                                                                                                                                                                                                                                   `aCGCCTATCTTAAAGTTTAAACATAAAGACCAGACCTAAAGACCAGACCTAAAGACACTACATAAAGACCAGAACTAAAGACGCCTTGTTGTTAGCCATAAAGTGATAACCTTTAATCATTGTCTGTAGTAATACAACTCACTATAAGGAGAGACAACTTAAAGAGACTTAAAAGATTAATTTAAAATTTATCAAAAAGAGTATTGACTTAAAGTCTAACCTATAGGATACTTACAGCCATCGAGAGGGACACGGCGAATAGCCATCCCAATCGACACCGGGGTCAACCGGATAAGTAGAc                         <  1:241907/301‑1 (MQ=255)`  
                                                                                                                                                                                                                                                                                                 `gTTTAAACATAAAGACCAGACCTAAAGACCAGACCTAAAGACACTACATAAAGACCAGACCTAAAGACGCCTTGTTGTTAGCCATAAAGTGATAACCTTTAATCATTGTCTTTATTAATACAACTCAATATAAGGAGAGACAACTTAAAGAGACTTAAAAGATTAATTTAAAATTTATCAAAAAGAGTATTGACTTAAAGTCTAACCTATAGGATACTTACAGCCATCGAGAGGGACACGGCGAATAGCCATCCCAATCGACACCGGGGTCAACCGGATAAGTAGActgtctcttatacac           >  1:100040/1‑287 (MQ=255)`  
                                                                                                                                                                                                                                                                                                        `cATAAAGACCAGACCTAAAGACCAGACCTAAAGACACTACATAAAGACCAGACCTAAAGACGCCTTGTTGTTAGCCATAAAGTGATAACCTTTAATCATTGTCTTTATTAATACAACTCACTATAAGGAGAGACAACTTAAAGAGACTTAAAAGATTAATTTAAAATTTATCAAAAAGAGTATTGACTTAAAGTCTAACCTATAGGATACTTACAGCCATCGAGAGGGACACGGCGAATAGCCATCCCAATCGACACCGGGGTCAACCGGATAAGTAGACAGCCTGATAAGTCGCACGaaa    >  1:161906/1‑301 (MQ=255)`  

                                                                                                                                                                                                                                                                                                          `|`                                                                                                                                                                                                                                                                                                              
`‑‑‑‑‑‑‑‑‑‑‑‑‑TCTCACAGTGTACGGACCTAAAGTTCCCCCATAGGGGGTACCTAAAG‑CCCAGCCAATCACCTAAAGTCAACCTTCGGTTGACCTTGAGGGTT‑CCCTAAGGGTTGGGGATGACCCTTGGGTTTGTCTTTGGGTGTTACCTTGAGTGTCTCTCTGTGT‑CCC‑TATCTGTTACAGTCTCCTAAAGTATCCTCCTAAAGTCACCTCCTAACGTCCATCCTAAAGCCAACACCTAAAGCCTACACCTAAAGACCCATCAAGTCAACGCCTATCTTAAAGTTTAAACATAAAGACCAGACCTAAAGACCAGACCTAAAGACACTACATAAAGACCAGACCTAAAGACGCCTTGTTGTTAGCCATAAAGTGATAACCTTTAATCATTGTCTTTATTAATACAACTCACTATAAGGAGAGACAACTTAAAGAGACTTAAAAGATTAATTTAAAATTTATCAAAAAGAGTATTGACTTAAAGTCTAACCTATAGGATACTTACAGCCATCGAGAGGGACACGGCGAATAGCCATCCCAATCGACACCGGGGTCAACCGGATAAGTAGACAGCCTGATAAGTCGCACGAAAAA  >  NC_001604.1/1‑582`

  

Alignment Legend

Aligned base mismatch/match (shaded by quality score): `ATCG/ATCG` `< 3 ≤` `ATCG/ATCG` `< 13 ≤` `ATCG/ATCG` `< 26 ≤` `ATCG/ATCG` `< 36 ≤` `ATCG/ATCG` `< 38 ≤` `ATCG/ATCG`

Unaligned base: `atcg`    Masked matching base: `atcg`    Alignment gap: `‑`    Deleted base: `‑`

Reads not counted as support for junction

read\_name Not counted due to insufficient overlap past the breakpoint.

read\_name Not counted due to not crossing MOB target site duplication.
